# Ethnicity and Pathway Progression: A Retrospective Cohort Study of Male Offenders Managed Under London Offender Personality Disorder Pathway Services

**DOI:** 10.1002/cbm.70044

**Published:** 2026-07-07

**Authors:** Roxanna Short, Sofia Turrientes Cordero, Philip Minoudis, Darrick Jolliffe, Mandip Brar, Jake Shaw, Colin Campbell

**Affiliations:** ^1^ Department of Forensic and Neurodevelopmental Sciences, IoPPN King’s College London London UK; ^2^ South London and Maudsley NHS Foundation Trust London UK; ^3^ Oxleas NHS Foundation Trust London UK; ^4^ Department of Security and Crime Science University College London London UK; ^5^ East London NHS Foundation Trust London UK

## Abstract

**Background:**

In the United Kingdom, the Offender Personality Disorder (OPD) Pathway provides a psychologically informed pathway of services—from screening to formulation to referral to treatments/interventions—for men and women whose offending is linked to their complex mental health needs. Evidence shows that individuals from most ethnic minority groups in the United Kingdom are over‐represented at all stages of the criminal justice system (including arrests, cautions and convictions). Such individuals may, however, be disadvantaged in terms of their access to and engagement with this specialist service.

**Aim:**

To examine the extent to which self‐reported ethnicity was associated with progression through discrete stages of the OPD Pathway in London.

**Methods:**

Linked administrative data from 8816 men who screened into the OPD Pathway from 2011 to 2022 were used to examine the association between self‐reported ethnicity (categorised as White, Black, Asian or mixed) and the last known recorded Pathway stage (screened‐in and no further work, case consultation/formulation, referral to services, accessing service and completion).

**Results:**

Adjusting for baseline risk, time on the OPD Pathway and London borough, we found that those who self‐identified as Black (compared to White) were under‐represented at all stages of the Pathway (compared to baseline), suggesting that these individuals tended to get ‘stuck’ at the early Pathway stages.

**Conclusions:**

These findings add weight to suggestions of probably disproportionate disadvantage among men identifying as Black in accessing important dedicated services for offenders with personality disorder. Our data have been used to inform exploration of programme changes, and next steps will include exploration of what has driven this under‐representation of non‐White ethnic groups across the service and whether more sensitively informed strategies can improve equitable access and benefits.

## 1 Introduction

Individuals with personality difficulties are disproportionately responsible for violent and serious offences (Craissati et al. 2020). We have used the term ‘personality difficulties’ here and will return to it, partly because Craissati does and partly because many people find the term personality disorder pejorative. Nevertheless, we acknowledge that it is a term widely accepted in the diagnostic and statistical manuals and used to plan services; a useful compromise was reached by the Personality Disorder Commission, informed by people with such difficulties, in ‘people who have been given a diagnosis of personality disorder’ (Lamb et al. 2018). To mitigate the risk of reoffending and protect the public, NHS England and His Majesty's Prison and Probation Service (HMPPS) jointly developed the Offender Personality Disorder (OPD) Pathway. This pathway provides psychologically informed services for individuals who have highly complex and challenging problems, are likely to have a personality disorder and are in prison and under probation supervision and who pose a high risk of harm to the public (NHS England 2023). It consists of multiple services in custody and the community for men and women with a likely diagnosis of personality disorder who meet defined risk criteria and for whom there is a functional link between the two. A formal diagnosis of personality disorder is not required; risk is assessed using the HMPPS Offender Assessment System (OASys) OPD Pathway screen (Minoudis and Shaw 2018). The OPD service in the community can be broadly split into indirect working or core offender management services, consisting of case identification, case consultation/formulation, a pathway plan and a referral to other services where appropriate; and direct community interventions (Intensive Intervention and Risk Management Services and often residence in Psychologically Informed Planned Environments (PIPEs) in Approved Premises) (NHS England 2023). Services in custody include treatment services and PIPEs at all levels of security. In PIPEs, the focus can be on preparation for treatment, optimising treatment engagement and delivery and consolidation of treatment gains (preparation, provision and progression PIPEs, respectively). Individuals always enter the Pathway through case identification and consultation and may remain solely under core offender management services. Progression to further stages in the Pathway is individualised and may not be linear, with a range of factors that may influence progression, such as availability of services and the stage of the individual's progression.

It has been demonstrated that individuals from minoritised ethnic backgrounds are more likely than those from White backgrounds to be stopped and searched, arrested, charged and remanded in custody (Lammy 2017; Ministry of Justice 2025), extending to an over‐representation of some groups, particularly those from Black and mixed backgrounds, across prison and probation services (27% of the UK prison population compared to 15% in the general population—HM Inspectorate of Prisons 2020; Lammy 2017). Despite their over‐representation at the point of entering the system, people from such minority groups appear less likely to engage in the support available in prison (Craissati et al. 2020). Some studies have highlighted the unique barriers facing them at each different point of access to support, which include their difficulties trusting staff and beliefs that the treatments available do not adequately reflect their needs (HM Inspectorate of Prisons 2020).

People from minoritised ethnic backgrounds also experience inequalities when accessing mental health services in the United Kingdom, experiencing longer waits for assessment and being less likely to receive a course of treatment (National Collaborating Centre for Mental Health 2023). Personality difficulties present specific challenges, characterised by pervasiveness (influencing multiple life domains) and chronicity (experienced over many years). Census data indicates that those meeting the criteria for personality disorders are about equally prevalent among White individuals and non‐White individuals across the United Kingdom (Lamph et al. 2023; Tyrer et al. 2015), but those from minoritised ethnic groups are less likely to be referred to, and to access, specialist services (Leese et al. 2006; Shingler and Pope 2018). Previous research suggests that people from minoritised ethnic groups were proportionately represented at most stages of the OPD Pathway in London but were significantly less likely to progress to the stage of receiving intervention services (Jolliffe et al. 2017). This suggests that there may be specific barriers to their progression within OPD Pathway services. Furthermore, this study is quite dated (using data from more than a decade ago) and was conducted during the first 48 months of the implementation of the Pathway when this unique approach to those with personality difficulties was still bedding in. As such, this finding may not accurately reflect broad changes or improvements in the Pathway or specific changes to resolve this disproportionality spurred by an increased awareness of some systemic barriers to some ethnic groups (NHS England 2023). Despite the critical importance of this issue, limited research exists on how ethnicity may be related to access to, or progression through, the OPD Pathway and also on what mechanisms may be driving any ethnic disparities (for an exception, see Hunter et al. 2019). This is of critical importance to the OPD Pathway in London, which operates in one of the most ethnically diverse areas of the United Kingdom, with recent census data indicating that 46.2% of London's population are from backgrounds other than White, indigenous backgrounds (Ministry of Justice 2025; Office for National Statistics 2022).

Our main aim, therefore, was to examine the ethnic distribution of men screened into the OPD Pathway in London and to observe the relationship between ethnicity and progression through the different stages of the Pathway.

## 2 Methods

### 2.1 Ethical Approval

This evaluation forms part of a wider study approved by the HMPPS National Research Committee (Reference: 2020‐002).

### 2.2 Sample and Data Source

This sample represents individuals who had accessed London OPD Pathway services from their inception (1 April 2011) to 1 September 2022. Individuals are screened onto the OPD Pathway via the Offender Assessment System (OASys), which is an actuarial risk and needs assessment completed by probation staff once an individual has been convicted of a criminal offence. Part of the OASys includes a personality disorder screening tool—the OASys personality disorder screen—and individuals scoring above a threshold of five are automatically screened onto the OPD Pathway. This assessment automatically updates the National *Delius*, which is the case‐management database used by probation services, and flags the individual as an OPD‐screened‐in case. Using this flag enabled us to identify each individual by their unique case identifier only and follow their data through automated data reports, also identified only by this unique number. These data reports yield descriptors of the individual's case (including sentencing, demographic, risk and case‐management data such as location and case status) and details of any contacts or interactions with the OPD Pathway services. For the study reported here, we accessed the reports on 1 September 2022 and identified *N* = 10,839 individuals who were flagged as either current or historic cases. We opted to remove women from the analysis due to their relatively low numbers (*N* = 696). We also excluded cases without an OPD contact or case status (*N* = 629) and those with missing, ‘unknown’ or ‘other’ ethnicity (*N* = 687). A further 11 individuals were excluded because their screening dates were before the OPD Pathway was established in 2011 and thus were likely to be reporting errors. This left a final sample of *N* = 8816.

### 2.3 Primary Outcome: Pathway Stage

The primary outcome of interest was the most recently recorded stage of the Pathway. Using data from either the most recent OPD contact type or case status, a person's stage was categorised as follows: (1) screened in, but no further work (reflected by case status descriptions of ‘pending consultation’ or ‘pending formulation’ and no further OPD contacts); (2) case formulation or case consultation; (3) referral to services; (4) accessing services; and (5) completion. For the purposes of the analysis, the reference stage (i.e., the stage used as the baseline comparison in the analyses) was ‘screened in’.

### 2.4 Covariates

Our primary exposure, self‐reported ethnicity (categorised as White, Black, Asian and mixed), was extracted from the Delius reports. These categories were grouped for analysis and reflect the broader ethnicity categories used in HMPPS reporting. Those categorised as Asian included those from Indian, Pakistani, Bangladeshi or ‘other’ Asian backgrounds. Individuals self‐reporting as ‘Asian or Asian British: Chinese’ were recoded as missing due to their small number (*N* = 15). Individuals self‐reporting as ‘Arab’ were also recoded as missing due to their small number (*N* = 50). Age in years, time on the Pathway (calculated as the days from referral to the date from which the Pathway stage was recorded—i.e., either the most recent contact or case status update), 1‐year Offender Group Reconviction Scale (OGRS; Howard et al. 2009) and location were extracted from Delius. The OGRS is an actuarial risk prediction tool for indicating the likelihood of a proven reoffence within 1 year of the assessment based on age, sex, index offence and criminal history. This tool has been evidenced as predictively valid and superior or equivalent to other actuarial risk assessment tools (Farrington et al. 2008).

### 2.5 Statistical Analysis

We examined the associations between ethnicity (Black, mixed and Asian vs. White) and Pathway stage (consultation/formulation, referral, accessing services and completion vs. screened in) using multinomial logistic regression on the complete‐case sample (*N* = 8816), estimating robust standard errors allowing for intragroup correlations relating to location and adjusting for OGRS score and time on the Pathway. Because there were five possible outcomes, we modelled the data with a multinomial distribution. The estimates from such models are expressed as relative risk ratios (RRRs), alongside their 95% confidence intervals (CIs). For a categorical predictor variable (i.e., ethnicity), this represents the change in relative risk for each level of the predictor, compared to its reference category, for achieving one of the outcomes compared to the baseline outcome, holding all other variables constant. Their interpretation is similar to that of an odds ratio, where an RRR of 1 means there is no difference/change in relative risk, an RRR of < 1 means there is a reduction in relative risk, and an RRR of > 1 means there is an increase in relative risk.

A large proportion of the sample was flagged as having spent time in custody at the time the reports were accessed (*N* = 3408, 39.8%). Because we did not have data on when the custody episode occurred (or how long it lasted), we opted, in addition, to conduct a sensitivity analysis adjusting for custody to assess whether this had a significant impact on the primary association of interest. There appeared to be differential missingness of ethnicity data by Pathway stage (12.5% missing at screening, 6% at consultation/formulation, 3% at referral, 5% at accessing services and 5% at completion), which was likely due to records being updated after work had been completed. As a result, we conducted additional sensitivity analyses using multiple imputation. We imputed missing ethnicity using multinomial logistic regression with age, OGRS score, location and time on the Pathway as predictors. We generated 20 imputed datasets using chained equations (White et al. 2011), ran the adjusted multinomial regression on each and pooled the results using Rubin's rules (Rubin 1987).

## 3 Results

Our final sample included *N* = 8816 men, with a median age of 37 (IQR 30–47), who were screened into the OPD Pathway in London. Of these, *N* = 403 (4.6%) were under the care of the Intensive Interventions and Risk Management Services. Forty‐two per cent (*N* = 3710) of the sample were White, 40% were Black (*N* = 3532), 10% were mixed (*N* = 990), and 8% were Asian (*N* = 694) (see Table 1 for details). Two thirds of the sample were recorded as being in the early consultation or formulation stages of the Pathway (*N* = 5877), and this was similar for each ethnicity. White men appeared to be under‐represented at the screened‐in stage of the Pathway and over‐represented at later Pathway stages (see Table 1).

**TABLE 1:** Pathway stage and offender demographics, by ethnicity (*N* = 8816).

	White	Black	Mixed	Asian	Total
	*N* (%)	*N* (%)	*N* (%)	*N* (%)	*N* (%)
Pathway stage					
Screened in	711 (19.2)	771 (21.8)	185 (21.0)	152 (21.9)	1819 (20.6)
Consultation or formulation	658 (17.7)	600 (17.0)	162 (18.4)	127 (18.3)	1547 (17.5)
Referral to services	1813 (48.9)	1752 (49.6)	425 (48.3)	340 (49.0)	4330 (49.1)
Accessing services	142 (3.8)	129 (3.7)	32 (3.6)	24 (3.5)	327 (3.7)
Completion	290 (7.8)	231 (6.5)	62 (7.0)	43 (6.2)	626 (7.1)
Age					
18–29	577 (15.6)	1023 (29.0)	317 (36.0)	201 (29.0)	2118 (24.0)
30–39	1174 (31.6)	1262 (35.7)	341 (38.8)	262 (37.8)	3039 (34.5)
40–49	868 (23.4)	635 (18.0)	129 (14.7)	149 (21.5)	1781 (20.2)
50–59	637 (17.2)	415 (11.7)	72 (8.2)	53 (7.6)	1177 (13.4)
*Missing*	454 (12.2)	197 (5.6)	21 (2.4)	29 (4.2)	701 (8.0)
OGRS score					
Median (IQR)	36 (16–55)	36 (21–52)	44 (28–58)	32 (15–51)	37 (20–54)
Time on the Pathway (months)					
Median (IQR)	41 (15–83)	45 (18–86)	38 (12–76)	43 (16–82)	37 (16–83)

Abbreviation: IQR, interquartile range.

**FIGURE 1:**
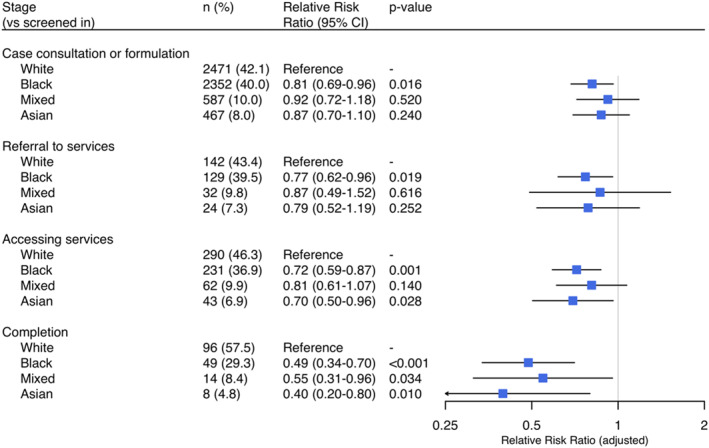
Forest plot showing the adjusted associations between ethnicity and Pathway stage.

Controlling for risk and time on the Pathway, this analysis showed that Black (vs. White) individuals were less likely to be represented at each stage of the Pathway (vs. screened in; see Figure 1). In addition, there appeared to be a dose–response‐type effect, with the relative risk ratio (RRR) increasing in magnitude from case consultation/formulation (vs. screened in; RRR = 0.81, 95% CI 0.69–0.96, *p* = 0.016), to referral to services (vs. screened in; RRR = 0.77, 95% CI 0.69–0.96, *p* = 0.019), to accessing services (vs. screened in; RRR = 0.72, 95% CI 0.59–0.87, *p* < 0.001), to completion (vs. screened in; RRR = 0.49, 95% CI 0.34–0.70, *p* < 0.001; see also Figure 1). Asian (vs. White) offenders were also under‐represented at the accessing services stage (vs. screened in; RRR = 0.70, 95% CI 0.50–0.96, *p* = 0.028) and the completion stage of the Pathway (vs. screened in; RRR = 0.40, 95% CI 0.20–0.80, *p* = 0.010). Mixed (vs. White) individuals were under‐represented at the completion stage of the Pathway (vs. screened in; RRR = 0.55, 95% CI 0.31–0.96, *p* = 0.034). Our sensitivity analysis indicated that further adjusting for the presence of a custodial episode did not affect these associations (see Supporting Information S1: Table S1). The multiple imputation sensitivity analysis produced results consistent with the complete‐case analysis (see Supporting Information S1: Table S2).

## 4 Discussion

Our analysis of these data shows that the majority of men screened into the OPD Pathway in London do appear to progress, at least in the early stages of the Pathway, which is promising. This is evidence that the early stage of the Pathway is operating as intended. There was also evidence, however, that, even at this early stage, Black men were under‐represented compared to their White peers and that this remained true at every subsequent stage of the OPD Pathway.

These findings add to previous research on the London OPD Pathway, not only by confirming in a larger complete‐cohort study that minoritised ethnic groups are under‐represented as the Pathway progresses but also by suggesting that compared to earlier figures (Jolliffe et al. 2017), their inclusion at later stages is probably decreasing. This pattern of service use also accords with patterns of under‐representation of ethnic minority groups reported in other forensic mental health services (Lamph et al. 2023; Leese et al. 2006; Shingler and Pope 2018). These results may point to wider systemic barriers that contribute to reducing service referrals or engagement for people in minority groups.

Approaches to engaging those from any minoritised ethnic group, but particularly those self‐defining as Black, should be culturally informed. Since the Lammy Report (2017), there has been an increased awareness of the pervasiveness and impact of disproportionality in the criminal justice system for those from Black and other non‐White backgrounds. Individuals from these backgrounds experience systemic discrimination from other social institutions too, including schools, housing and health services, which, in turn, increases their exposure to many risk factors associated with a host of negative outcomes (e.g., Iruka et al. 2022). More specifically, work by Uhrig (2016) found that policing and court decision‐making tended to be biased against those from Black, Asian and other minoritised ethnic groups. In addition, their experiences of probation and prison tended to be more negative than those of their White peers (e.g., Uhrig 2016). Previous research indicated that, in stepped pathways in criminal justice services, biases emerge at points where discretion in decision‐making can be used by professionals (Lammy 2017; Lamph et al. 2023; Shingler and Pope 2018). These experiences of discrimination pose very serious challenges for engagement with relevant services, particularly in the criminal justice system (e.g., Rashid 2022). In addition, people on probation from minoritised ethnic groups may be less likely to engage with voluntary OPD Pathway services, perhaps due to individual or systemic experiences of discrimination in the criminal justice system (Shingler and Pope 2018). Although a number of promising culturally aware and informed approaches have been proffered (e.g., Wright and Williams 2015), it is clear that much more evidence on effective working with those from these backgrounds is needed (User Voice 2023). Based on the results from our study, it is not possible to determine the drivers of these inequalities, but this is a very important topic for future research. Some co‐production work has already been completed and is promising (Health Service Journal 2023).

The strengths of this evaluation include the large dataset with clearly delineated Pathway steps and a statistical modelling approach that allowed for the observation of the relationship between ethnicity and Pathway stages, controlling for alternative explanations (i.e., age, risk and time on the Pathway). The primary limitation is the quality and coverage of the available data. These are routinely collected data drawn from a number of different statistical reports and case‐management databases, which may be impacted by variation in reporting practices between probation teams in London. Furthermore, we did not have access to data relating to external services to which some people on probation may have been referred. This may explain why a substantially smaller proportion of the sample was recorded as having reached the accessing services and completion stages of the Pathway. Although there were substantial missing data on self‐reported ethnicity that appeared to be related to Pathway stage, our findings were robust according to multiple imputations of missing ethnicity using strong auxiliary predictors, suggesting that differential missingness is unlikely to explain the observed ethnic disparities in Pathway progression. A further limitation is that these are cross‐sectional data, so we cannot draw conclusions about whether ethnicity is associated with the rate of progression through the Pathway. Future research should consider a longitudinal approach to examine Pathway progression, but this may be hindered by the nature and availability of the data. As there are significant regional variations in the distribution of different ethnicities, it would be useful to replicate these analyses at a national level to examine whether these disparities are reflected in similar OPD Pathway services across England and Wales.

## 5 Conclusions

This research offers insights into ethnic disparities at each stage of delivery of the Offender Personality Disorder Pathway in London, a service for mitigating the risk of reoffending by individuals who have highly complex and challenging problems and are likely to have a personality disorder, and thereby protecting the public and promoting the health and (re)habilitation of offenders. It suggests the need for clinically or socially significant service adaptations and further investigation into the factors driving the under‐representation at each stage. London is a multicultural city with great opportunities for resolving such disparities. Our findings have already been made available to relevant services in London and, we understand, have been used to inform a quality‐improvement (QI) approach to reducing these disparities, using process mapping to identify key decision points where disproportionalities occur and implementing targeted adaptations. Community OPD services in London, for example, have introduced a structured checklist to support practitioners in identifying culturally appropriate and perhaps specifically designed referral options, and in at least one prison‐based PIPE service a co‐produced programme of work has been undertaken to make referral processes more culturally responsive. Next steps will be the analysis of new cohorts of OPD Pathway users, using our analyses as reference points.

## Funding

The authors have nothing to report.

## Conflicts of Interest

The authors declare no conflicts of interest.

## Supporting information


Supporting Information S1

## Data Availability

The data relating to this study are available from the National Applications Reporting Team following HMPPS National Research Committee approval and data‐sharing agreements.
