# Supplementary material for: Ethnicity and Pathway Progression: A Retrospective Cohort Study of Male Offenders Managed Under London Offender Personality Disorder Pathway Services
Source: Crim Behav Ment Health. 2026 Jul 7;36(4):173–8. doi: 10.1002/cbm.70044 (PMC13432926; doi:10.1002/cbm.70044)
Supplement: Supplementary file 1 — Supporting Information S1 [file CBM-36-173-s001.docx]

# Supplementary Materials

Table S1: Results of the adjusted multinomial logistic regression examining the association between ethnicity and pathway stage

|  |  | aRRR (95%CI) | p |
| --- | --- | --- | --- |
| Screened in | | (baseline outcome) | |
|  |  |  |  |
| Consultation/formulation | | |  |
| Ethnicity |  |  |  |
|  | White | (reference) | |
|  | Black | 0.83 (0.70-0.99) | 0.037 |
|  | Mixed | 0.95 (0.74-1.22) | 0.683 |
|  | Asian | 0.87 (0.70-1.10) | 0.244 |
| Referral to services | | |  |
| Ethnicity |  |  |  |
|  | White | (reference) | |
|  | Black | 0.79 (0.63-0.99) | 0.042 |
|  | Mixed | 0.89 (0.51-1.58) | 0.697 |
|  | Asian | 0.79 (0.52-1.19) | 0.255 |
| Accessing services | |  |  |
| Ethnicity |  |  |  |
|  | White | (reference) | |
|  | Black | 0.74 (0.60-0.90) | 0.003 |
|  | Mixed | 0.83 (0.63-1.11) | 0.220 |
|  | Asian | 0.70 (0.51-0.96) | 0.028 |
| Completion | |  |  |
| Ethnicity |  |  |  |
|  | White | (reference) | |
|  | Black | 0.52 (0.36-0.75) | 0.001 |
|  | Mixed | 0.60 (0.34-1.06) | 0.079 |
|  | Asian | 0.40 (0.21-0.79) | 0.008 |
| Note: aRRR = adjusted relative risk ratio. Adjusted for risk, time on pathway, and presence of a custodial episode. Standard errors are adjusted for clustering effect of offender manager team location. | | | |

Table S2: Comparison of results from the complete-case analysis and the multiple imputation analysis.

|  |  | Complete-case (N=8,916) | | Multiple Imputation (N=9,502) | |
| --- | --- | --- | --- | --- | --- |
| Stage (vs screened in) | Ethnicity (vs white) | aRRR (95% CI) | p | aRRR (95% CI) | p |
|  |  |  |  |  |  |
| Consultation/formulation | |  |  |  |  |
|  | Black | 0.81 (0.69-0.96) | 0.016 | 0.82 (0.69-0.96) | 0.014 |
|  | Mixed | 0.92 (0.72-1.18) | 0.520 | 0.90 (0.71-1.15) | 0.416 |
|  | Asian | 0.87 (0.70-1.10) | 0.240 | 0.86 (0.69-1.07) | 0.183 |
|  |  |  |  |  |  |
| Referral to services | |  |  |  |  |
|  | Black | 0.77 (0.62-0.96) | 0.019 | 0.77 (0.62-0.96) | 0.020 |
|  | Mixed | 0.87 (0.49-1.52) | 0.616 | 0.84 (0.49-1.46) | 0.546 |
|  | Asian | 0.79 (0.52-1.19) | 0.252 | 0.78 (0.52-1.18) | 0.235 |
|  |  |  |  |  |  |
| Accessing services | |  |  |  |  |
|  | Black | 0.72 (0.59-0.87) | 0.001 | 0.72 (0.59-0.87) | 0.001 |
|  | Mixed | 0.81 (0.61-1.07) | 0.140 | 0.79 (0.59-1.05) | 0.101 |
|  | Asian | 0.70 (0.50-0.96) | 0.028 | 0.68 (0.47-0.96) | 0.029 |
|  |  |  |  |  |  |
| Completion |  |  |  |  |  |
|  | Black | 0.49 (0.34-0.70) | <0.001 | 0.49 (0.34-0.72) | <0.001 |
|  | Mixed | 0.55 (0.31-0.96) | 0.034 | 0.56 (0.32-0.98) | 0.043 |
|  | Asian | 0.40 (0.20-0.80) | 0.010 | 0.39 (0.19-0.80) | 0.011 |
| Note: aRRR = adjusted relative risk ratio. Adjusted for risk, time on pathway, and presence of a custodial episode. Standard errors are adjusted for clustering effect of offender manager team location. | | | | | |
